# A Mixed-Methods Systematic Review of the Impacts of Coronavirus on Society and Culture

**DOI:** 10.3390/ijerph18020491

**Published:** 2021-01-09

**Authors:** Yeon Jung Yu, Young Su Park, Alison Keller, Jin-Won Noh, Jiho Cha

**Affiliations:** 1 Department of Anthropology, Western Washington University, Bellingham, WA 98225, USA; Yuy2@wwu.edu (Y.J.Y.); kellera5@wwu.edu (A.K.); 2 Center for the Arts and Humanities, Haverford College, Haverford, PA 19041, USA; ypark3@haverford.edu; 3 Department of Health Administration, Dankook University, 119, Dandae-ro, Dongnam-gu, Cheonan-si, Chungcheongnam-do 31116, Korea; 4 Humanitarian and Conflict Response Institute, University of Manchester, Manchester M13 9PL, UK; jiho.cha@manchester.ac.uk

**Keywords:** COVID-19, discrimination, stigma, culture, society

## Abstract

Little is understood of the social and cultural effects of coronaviruses such as coronavirus disease 2019 (COVID-19), severe acute respiratory syndrome (SARS) and Middle East respiratory syndrome (MERS-CoV). This systematic review aims to synthesize existing findings (both qualitative and quantitative) that focus on the social and cultural impacts of coronaviruses in order to gain a better understanding of the COVID-19 pandemic. Utilizing a predetermined search strategy, we searched CINAHL, PsycINFO, PubMed, and Web of Science to identify existing (qualitative, quantitative, and mixed-methods) studies pertaining to the coronavirus infections and their intersection with societies and cultures. A narrative synthesis approach was applied to summarize and interpret findings of the study. Stemming from SARS outbreak in 2003, qualitative and quantitative findings (twelve adopted quantitative methods and eight exclusively used qualitative methods) were organized under five topical domains: governance, crisis communication and public knowledge, stigma and discrimination, social compliance of preventive measures, and the social experience of health workers. The selected studies suggest that current societies are not equipped for effective coronavirus response and control. This mixed-methods systematic review demonstrates that the effects of coronaviruses on a society can be debilitating.

## 1. Introduction

This review examines the existing literature regarding coronavirus diseases and their intersection with society and culture. Coronaviruses, including severe acute respiratory syndrome (SARS) and Middle East respiratory syndrome (MERS-CoV), have had large effects on the world during and after the outbreaks. Given the contemporary global pandemic of coronavirus disease 2019 (COVID-19), it is important to understand the effects of pandemics/epidemics on society and culture. Specifically, this review intends to identify topics, the scope of existing research, and the knowledge gap in studies of coronaviruses and society/culture and provide a direction for future research in the field. For working definitions for this review, culture is a shared understanding that constitute practices and make them meaningful [1], and as such includes issues of stigma, knowledge, beliefs, compliances and media representations, while societal dimensions of epidemics deal with larger structural conditions that shape governance and social inequalities.

In November 2002, severe acute respiratory syndrome (SARS) originated in the Guangdong providence in China [2]. Symptoms of SARS include a fever of over 100.41° F, dry cough, diarrhea, vomiting, and pneumonia, and the virus is spread though close contact [3]. In Hong Kong, SARS infected 1755 citizens and 299 lost their lives [4]. In February 2003, SARS was brought to Toronto, Canada, by a traveler from Hong Kong [2]. Overall, there were 8069 cases and 774 deaths in China, Taiwan had 251 cases, Singapore has 238 cases, and Toronto had 251 cases [5].

Victims of SARS faced stigmatization before and after the SARS outbreak [4]. SARS caused high levels of fear in many places around the world, stemming from the lack of information about the deadly epidemic [3], which led to the “othering” and discrimination of Chinese people. The media also had a role in stigmatization by dramatizing the virus, blaming the sick for putting others at risk, promoting “othering”, and enforcing moral and political agendas [3]. Stigmatization extended to healthcare workers (HCWs) as well. In Toronto, resources were strained in hospitals as many healthcare workers were quarantined and others had to work extra shifts to manage the large patient loads [6]. During the SARS outbreak in Taiwan, 20% of healthcare workers experienced stigmatization from their communities; in Singapore, 49% of HCWs reported experiences of stigmatization [7].

The Middle East respiratory syndrome coronavirus (MERS-CoV) is a respiratory infection belonging to the genus Betacoronavirus that first appeared in Saudi Arabia in 2012 [7,8]. It is transmitted through respiratory droplets or close contact with infected people, and one-third of MERS-CoV patients die from the disease [9]. Symptoms of MERS-CoV include fever, coughing, respiratory problems, and sometimes diarrhea [9]. In May of 2015, MERS-CoV also spread to South Korea by an infected traveler from the Middle East [9]. In South Korea, as of July 2015, 186 people were infected, 36 of whom died, and 16,693 were quarantined as a preventative measure [9,10]. Forty-one Korean healthcare workers were infected with the virus, making up 22% of infected persons, and 40% were nurses [7,11]. Worldwide, there have been a total of 1374 infections and upwards of 490 deaths related to MERS-CoV [9].

During SARS and MERS, media was used to raise awareness, provide updates, and communicate preventative measures [9]. For example, media consumption increased during the outbreak in Hong Kong; television was used at a rate of 87%, and the internet followed with a rate of 71%, following the large media coverage [9]. Media also caused concern for mass gatherings. The transmission of infectious diseases such as MERS-CoV is high during large gatherings, and the Hajj pilgrimage in Mecca, Saudi Arabia was a large concern because of the public health challenge it created during the outbreak [12].

Since the SARS outbreak in 2003, researchers have conducted studies on the spread of coronaviruses, the characteristics of the infections, and how these outbreaks have impacted public health. The epidemiological evidence of coronaviruses was synthesized with a systematic literature review, which identified incubation period, reproductive numbers, doubling time, as well as the fatality rate of COVID-19 [13]. A systematic literature review investigated the prevalence of comorbidities with MERS-CoV and found that diabetes and hypertension were equally prevalent in 50% of 637 MERS-CoV cases [14]. Another systematic literature review examined literature on the safety and efficiency of therapies for SARS patients using lopinavir/ritonavir, convalescent plasma, interferon alpha, and ribavirin therapy, finding that ribavirin may improve and reduce mortality [15]. In addition, a literature review on viral and bacterial infectious diseases that focuses on mass gatherings found infectious disease prevalence continued to increase during Hajj, but no cases of MERS-CoV have been identified in Hajj pilgrims [16]. Similarly, a systematic literature review regarding the uptake and effectiveness of facemasks during mass gatherings resulted in a stated 53.5% average uptake of facemasks, although the effectiveness of facemasks varied widely based on thirteen studies [17].

The contemporary COVID-19 crisis has a mixed nature of “coronavirus” and “pandemic”, although its magnitude as a pandemic is not comparable with those in the recent past such as SARS and MERS. In response to the COVID-19 pandemic, this mixed-method review aims to explore what lessons have been learned from previous coronavirus epidemics, especially societal experience of SARS and MERS. COVID-19 has not only reflected epidemiology, pathogenesis, and clinical characteristics as a novel coronavirus, but their social and cultural intersection as pandemics has also represented stigmatization, discrimination, politicization, and othering. To the best of our knowledge, the current study is the first attempt to look at the overall impact of coronaviruses on society and culture.

## 2. Methods

We have adopted a mixed-method systematic review to synthesize evidence from different methodological approaches of quantitative, qualitative and mixed-methods studies [18]. This mixed-methods approach is particularly evident to examine the comprehensive research question of coronavirus diseases and their intersection with society and culture [19].

### 2.1. Search Strategy and Selection Criteria

Empirical studies written in English from peer reviewed journals were systematically searched in four electronic bibliographic databases: CINAHL, PsycINFO, PubMed, and Web of Science, using terms combining two main components: (a) coronavirus, severe acute respiratory syndrome (SARS), Middle East respiratory syndrome (MERS); and (b) culture and society. Culture and society were defined broadly for the wider inclusion of findings concerning the social and cultural impacts of coronavirus. All findings from qualitative, quantitative, and mixed-methods were included. The literature search was conducted on 25 March 2020 and updated on 25 September 2020.

**Figure 1:**
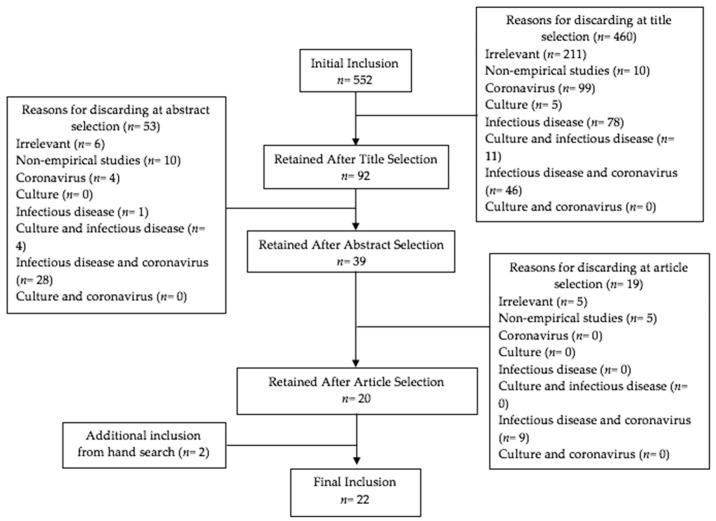
Flow diagram of study selection.

The initial search found 965 results. After deleting duplications, 552 sources remained to be further reviewed. We included studies that were: (1) peer-reviewed and published in English-language journals; (2) empirical studies using either qualitative or quantitative methodology; (3) focused on coronaviruses and their interaction with society and culture. Citations were screened using a four-step process that included title review, abstract review, article review, and hand search (see Figure 1). During title review, the unduplicated articles (*n* = 552) were screened to exclude citations that did not provide empirical data on coronaviruses and society. This eliminated 211 sources for irrelevance, 10 for being non-empirical, and 239 articles that focused on only one or two of our three primary topics of interest (i.e., infectious disease, coronavirus, society/culture). At the abstract review stage, of the remaining 92 sources, 53 were excluded that did not incorporate all three topics or were non-empirical studies. Finally, the remaining 39 sources were reviewed. At this stage, five sources were excluded for irrelevance, five sources were non-empirical, and nine only covered coronavirus or other infectious diseases. The 20 articles that remained after the screening covered the impact of coronavirus epidemics on society/culture. At the conclusion of this process, a hand search was conducted by reviewing the reference lists of those screened articles, and two additional articles were found.

### 2.2. Data Sources and Data Abstraction

The literature search was conducted on March 25, 2020, using four electronic bibliographic databases: CINAHL, PsycINFO, PubMed, and Web of Science, using the keywords “infectious disease”, “coronavirus” (alternatives search terms were SARS and MERS), and “culture” (alternative search term was society).

## 3. Results

### 3.1. Characteristics of the Reviewed Studies

#### 3.1.1. Study Sites and Publication Period

The key characteristics of the reviewed studies are summarized in Table 1 by author(s), publication year, study site, year of data collection, sample size, age of study sample, and study design. Data were collected from Canada (4), the U.S. (2), Australia (1), the United Kingdom (2), Saudi Arabia (2), South Korea (5), and China (3). One study was conducted in eight different countries (i.e., Denmark, The Netherlands, Poland, Spain, U.K., China, Hong Kong, and Singapore) [20] Countries that had outbreaks of coronaviruses tended to have more conducted studies. The reviewed articles were published between 2003–2020; 11 were published between 2003–2009 and 15 were published between 2014–2020. None of the studies were published between 2009 and 2014, which is likely because of the gap between outbreaks of SARS in 2003 and MERS-CoV in 2014.

#### 3.1.2. Study Design and Target Population

Out of the 22 articles, twelve adopted quantitative methods and eight exclusively used qualitative methods. Two studies did not fall into these categories [2,21]. The samples of these studies consisted of a large range of populations, from Hajj pilgrims, to healthcare workers, and to general members of the public. The range of the sample size also varied dramatically, from 14 to 3436, with a mean sample size of 580.3. One study did not include the number of people surveyed [22]. Four studies solely drew upon secondary sources, such as documents, media, and health statements [2,5,6,21].

The target population of the selected studies largely depended on the purpose of study, as illustrated in Table 1. For instance, studies focusing on mass gatherings targeted populations of Hajj pilgrims [12,23]; studies focusing on healthcare workers explored nurses and physicians [7,11]. The age of study populations varied as well, with the mean age being 30.5 years old (among six studies which specified the age information of participants). Five studies reported a sample age as 18 and above [8,9,20,24,25]. Ten studies did not include a specific age in their samples.

### 3.2. Theme

#### 3.2.1. Governance

Three studies focused on the theme of governance, adding to evidence that government intervention, or lack thereof, can drastically change the impact of the outbreak on society. One study focused on South Korea’s recently reviewed infectious disease procedure legislation and found the need for improvements dealing with all matters of infectious diseases and creating roles for each level of government and promoting collaboration between them [21]. By looking at the vulnerabilities of worldwide public health responses, another study found issues such as cultural and political conflicts, lack of personnel and resources, policy, and other issues demonstrating that the world was not prepared for a global pandemic [26]. A study that examined Canadian emergency plans concluded that there was a lack of support for healthcare workers during viral outbreaks [6]. These reviewed articles yield a general pattern of necessary improvements for government-based preparations for infectious disease outbreaks.

#### 3.2.2. Crisis Communication and Public Knowledge

Out of the selected studies, five articles focused on media and the ways in which communication, metaphors, and discourse can change public perceptions around the infections. By exploring the disease metaphors used in newspapers in the U.K. to describe SARS, a study found that the metaphor “killer” was heavily associated with the virus [5]. Another study found that mass media exposure increased preventative measures during the 2015 MERS outbreak through interpersonal communication after media exposure and increased concern about social environments, resulting in more prevention measures [9]. In a study in the United Kingdom and The Netherlands, media, including Chinese media and British/Dutch TV, was reported as the second most important source of information distribution on SARS [27]. In South Korea, social network sites were used to express and receive information by the public during the 2015 MERS outbreak, and the higher levels of MERS-CoV information that were received led to higher levels of personal prevention measures, such as hand washing and cough etiquette [10]. A similar study in South Korea also found that social media use during the MERS 2015 outbreak positively affected emotions of fear and anger, which positively related to the public risk perception [28]. The strong influence which the media has on both public knowledge and perception is suggested by these reviewed studies.

Five of the 22 studies examined public knowledge of coronaviruses, showing the variation in responses and awareness of each virus across the globe. For example, a study in Saudi Arabia found that higher concern of MERS-CoV led to more prevention measures being implemented, overall higher levels of proper hygienic practices, and found that knowledge was a significant predictor in the level of concern and preventative measures [8]. Another study in Australia found that, by looking at Hajj pilgrims and their preventative measures against MERS-CoV and other viruses, 94% of participates practiced hand hygiene, 53% used face masks, and those who received pre-travel advice were two times more likely to receive a vaccine [12]. The perceived threat of SARS and its severity was higher in Europe; both perceived vulnerability and response efficiency was found to be higher in Asia, and comparative vulnerability was linked with knowledge and gender [20]. In New York City, socioeconomic class, race, and ethnicity factored into individuals being less informed on SARS and AIDS and more concerned with contracting the diseases [24]. By exploring the sources of information of Chinese communities in the U.K. and The Netherlands, another study found that knowledge of SARS and avian flu was high in these communities, and the majority of information came from family and friends [27].

#### 3.2.3. Stigma and Discrimination

Stigmatization acted as an important theme, showing how blame and stereotypes were crafted around the spread of SARS and MERS. In one study in South Korea, social stigmatization was a main influence on emergency nurse’s ethical issues during the MERS-CoV outbreak, followed by the level of agreement with infection control measures and the perceived risks of infection [11]. Another study found that nurse’s mental health in South Korea was worse when faced with greater stigma, and that stigma had an indirect effect on mental health through stress during the MERS-CoV outbreak [7]. A study of U.K. newspaper coverage of SARS found that stigmatization of a certain place or culture was lower in the beginning of the SARS outbreak due to the lack of a localizing name, such as West Nile Virus; it was harder to pinpoint the blame on one place which most likely led to less racial, social, and national stigmatization [5]. In interviews in New York City’s Chinatown after SARS, the participants found themselves reaffirming stereotypes of Chinese culture and association with disease, while also directing the blame and stigma toward recent Chinese immigrants [3]. In Toronto, those who were quarantined during the SARS outbreak felt that they experienced stigmatization, rejection, scrutinization, and isolation both during and after quarantine [25]. Victims of SARS in Hong Kong faced social stigma enforced by medical professionals, government institutions, and the general public, years after the outbreak and their treatment [4].

Culture-specific experiences of coronaviruses were also found in the selected studies, providing case evidence of the differences that occurred in various responses in different countries. In Hong Kong, the capitalistic assumptions and values led 43% of SARS victims to resume work even though they all suffered degrees of stigmatization in the workplace [4]. Another unique experience reviewed is that of New York’s Chinatown, where there were no cases of SARS in the city, but stigma and discrimination towards Chinese people and recent immigrants was prevalent [3]. During the initial SARS outbreak in China, rumors and panic spread due to no official information being confirmed for months, which led to the panic buying of drugs that were rumored to prevent or control the virus [29].

Two reviewed studies examined social inequalities that affected issues surrounding coronaviruses and the ways in which inequalities can be exacerbated by a virus. In New York City, it was found that participants in lower socioeconomic groups—racial/ethnic minorities with lower formal education and lower income—were less likely to be informed about SARS (and AIDS) and were more likely to be worried about the contraction of both diseases [24]. A different study in New York City showed the “othering” of recent Chinese immigrants, tied to fear that they would spread SARS to New York’s Chinatown [3]. This “othering” reflected power struggles in the community as well as social, political, and economic inequalities [3].

#### 3.2.4. Social Compliance of Preventive Measures

Compliance to prevention measures was also examined in other studies in which compliance acted as an example of the successes of countries in responding to the virus across a variety of cultural settings. Both the Saudi Arabia and Australia studies reported frequent hand washing (94%) and facemask use; 74% of Saudis used facemasks and 81% avoided touching their face altogether [8]. For the Australians, 53% used facemask [12]. In South Korea, handwashing intention and cough etiquette were both high (4.25 and 4.24 out of 5) [10]. A study conducted in Mecca, Saudi Arabia, during Hajj, 2015, found high participation in prevention measures; people aged >35 maintained significantly better practices [23]. The uptake of prevention measures for Hajj participants, such as the use of facemasks and frequent hand washing, was a culture-specific experience during the MERS-CoV outbreak [12,23].

Three selected studies examined religious mass gatherings of the Hajj pilgrimage to Mecca, an important case study providing evidence of the changes in culture and society that coronaviruses can cause. One study found high prevention measures of Australian pilgrims during Hajj, 2014 [12]. Another study using surveillance data following travelers from the Middle East, including Hajj and Umrah pilgrims, found a small number of sporadic travel-associated MERS-CoV cases, whereas cases of influenza, rhinovirus, and other infectious diseases were common [22]. Finally, a study of Hajj pilgrims’ knowledge and attitudes toward MERS-CoV found that their knowledge of MERS-CoV was average but their compliance with prevention practice was high [23].

#### 3.2.5. Social Experience as Healthcare Workers

Out of the selected papers, five studies focused on healthcare workers and their experiences of coronavirus epidemics. Healthcare workers have a direct impact on coronavirus treatment, spread, and outcome; therefore, there was a considerable amount of research conducted on their experiences during SARS and MERS-CoV. One study found that there was little emotional and social support for healthcare workers in Canadian emergency plans, while instrumental and informational supports were predominant [6]. As mentioned above, emergency nurses’ ethical issues during the MERS-CoV outbreak in South Korea were influenced by social stigma, infection control measures, and perceived risk [11]. Another study in South Korea found that nurses’ mental health was directly and indirectly worsened by stigma and stress during the MERS-CoV outbreak [7]. A study conducted during the SARS outbreak in Toronto found physicians who interacted closely with SARS patients continued to demonstrate professionalism through caring for patients, accepting personal risk, respecting confidentiality, and being role models for junior physicians [30]. A study focused on Toronto’s healthcare system and the problems that were faced during the SARS outbreak in 2003 found a comprehensive disease control strategy for enhanced control measures while keeping in mind clinical uncertainty and the flow of human contacts during SARS [2].

## 4. Discussion

The current review has synthesized the existing literature and found that countries around the globe are facing various socio-cultural challenges in overcoming coronavirus epidemics. As the first systematic literature review focused on coronaviruses and the way they impact society, this paper has consolidated the emerging themes and findings, which could lead to recommendations for future research. As this review shows, there is still much to be understood regarding the impact of viral coronaviruses on different cultures and societies.

With extensive stigma, unprepared governments, inconsistent public knowledge, strained healthcare systems and workers, and social inequalities faced in certain communities, the effects of a coronavirus on society can be debilitating. Governments are in need of improvement in coronavirus response preparedness. This study found that, although both diseases had large impacts, SARS’ economic and social impact was larger because of the lack of government communication of confirmed information about the virus during the outbreak. Ten years after SARS, H7N9 had less of an impact because of the effectiveness of the Chinese government’s communication strategy [29]. A study of public opinion on South Korean government communication with the public during MERS-CoV found that there was high distrust in the government, which was increased by cynicism, anger, and anxiety; these feelings led to citizens taking action toward the government [31]. Another study conducted on public opinion in China in the early stage of the COVID-19 outbreak suggested that government response should be strengthened in terms of public opinion, epidemic prevention, and control in important epidemic areas [32]. In the case of the 2020 coronavirus pandemic, a government that provided effective response to COVID-19 was Vietnam’s. A study on Vietnam’s policy response found that their political readiness, timely communication of information, and cooperation between government and citizens led to an effective response [33,34]. Government preventive responses to mitigate unexpected health outcomes from COVID-19 control measures include specific plans to protect vulnerable populations, such as the elderly, who are most likely to suffer from physical and mental health crises and nutritional insufficiencies from institutional care and self-isolation [35,36]. These findings show a mixed result of government improvement, but still show gaps in governmental responses to viral outbreaks.

Furthermore, the media’s considerable influence on public knowledge and risk perception, as indicated by selected studies, shows potential inconsistencies in public understanding and its response to coronaviruses; the close relationship that public knowledge has with response, level of concern, and uptake of prevention measures becomes clear. As shown in studies examining mass gatherings, high prevention measures taken by pilgrims helped to prevent the spread of MERS-CoV [22,37]. Media bias has been a contemporary issue regarding COVID-19, as found in a study conducted on Chinese and American newspapers [38]. Similarly, social media exposure in China was found to have a positive association with mental health issues, such as depression and anxiety during the COVID-19 pandemic [39]. Therefore, effective media and increased public knowledge has the potential to make the response to coronaviruses more effective.

The findings of the selected studies on healthcare systems, stigma, and social inequalities corroborate the evidence that the world is currently unequipped to handle coronavirus outbreaks on a pandemic scale. Societies suffer from a lack of support systems rendering them unprepared to handle the stress, emotional and mental health problems, ethical issues, and economic complications [40,41]. The consistent instances of stigma found during both SARS and MERS-CoV confirm that existing cultural and structural issues can have a negative effect on the ability of a society to respond effectively to an outbreak. The current pandemic confirms these conclusions; similar stigma has been experienced during COVID-19 [42]. In addition, a study examining the ethical and legal issues following the national and international response to SARS exposed the social class divisions revealed by home quarantine, the racial discrimination of Chinese people in North America, and the social inequalities which quarantine and isolation cause [43]. Social inequalities that have exacerbated the impact of SARS and MERS-CoV show that structural inequalities need to be addressed as well in order to best prepare a country for responding to a pandemic.

There were some limitations in the current literature review that prevent a full picture of the field from developing. Firstly, non-English studies were excluded because of accessibility issues for a general audience. A certain amount of research was conducted in non-English speaking countries, such as China and Korea where the SARS and MERS-CoV epidemics were centered; thus, this may have prevented a body of work from being analyzed. Secondly, the novelty of the topic and the relevance in the face of the contemporary COVID-19 pandemic limits our ability to fully assess the impact of this literature review. While the majority of this research was conducted over the past fifteen years, its implications for the present COVID-19 pandemic cannot adequately be understood because the virus itself is still developing, as are the responses around the world. Thirdly, the fact that these studies cover research from across the globe and include multiple viruses means that our ability to compare the research directly is limited; many of the studies focus on very different locations, subjects, and research topics. In addition, the wide variety of research types, from participant interviews to surveys to content analysis, prevents a full analytical picture from developing. However, despite these limitations, the current systematic literature review provides an overview of the topics that have been researched regarding how coronaviruses have been experienced in different societies, as well as the repercussions that follow those experiences.

## 5. Conclusions

Given the limited number of studies and urgency of the topic, we suggest potential directions for future research involving coronaviruses and society/culture. The first possibility would be more in-depth qualitative studies in order to identify the social mechanisms of transmission, because this will yield useful insights for future interventions and policies. Identifying political determinants or ways that different governments react to the epidemics would contribute to deepening our understanding of the diversity of government responses over time, as well as our understanding of different cultures and societies. Studying both cultural modifications made in response to an outbreak as well as how cultures experience an outbreak differently will provide an important perspective on the epidemics. Another possibility is to examine what improvements have been made to epidemic responses; the prevention strategies include, but are not limited to: biotechnology, enforcement of prevention measures, and social solidarity. In addition, identifying the most disadvantaged subpopulations by adopting a “vulnerability assessment tool” would be useful in addressing social inequalities and unequally affected populations in various societies. Furthermore, studies need to be conducted to identify the adjusted social networks that facilitate, or suppress, coronavirus spread under the social distancing policies and guidelines. By taking these directions, we could contribute to the development of prevention theory by constructing risk profiles and identifying the impacts of social networks. Such examination of epidemics should be comprehensive, including behavioral, biomedical, and structural approaches. Given the current COVID-19 pandemic, it would also be necessary to conduct systematic mixed-methods studies on various critical issues, such as culture-specific perceptions of COVID-19, modification of cultural/religious practices, local compliance to public health guidelines, enforcement of government orders, biotechnology, healthcare and services, social stigma, and social inequalities throughout the duration of the pandemic.

## Figures and Tables

**Table 1:** Summary of results with themes.

Authors (Year)	Ref #	Theme 1 Governance	Theme 2 Communication and Public Knowledge	Theme 3 Stigma and Discrimination	Theme 4 Social Compliance	Theme 5 Health Workers	Study Site	Study Sample and Size	Study Design
O’Sullivan et al. (2007)	6	Governance				Health Workers	Canada	12 emergency plans	Qualitative content analysis
Almutairi et al. (2015)	8		Communication and Public Knowledge		Social Compliance		Riyadh, Saudi Arabia	1147 male and female Saudis and expatriates 18 years and older in shopping malls	Quantitative Cross-sectional
Wallis & Nerlich (2005)	5		Communication and Public Knowledge	Stigma and discrimination			United Kingdom	5 UK newspapers	Qualitative linguistic method
Alqahtani et al. (2016)	12		Communication and Public Knowledge		Social Compliance		Australia	356 Australian Hajj pilgrims	Quantitative Two Cross-sectional surveys
Choi & Kim (2018)	11			Stigma and discrimination		Health Workers	South Korea (3 cities)	169 emergency nurses	Quantitative Cross-sectional
Park (2017)	21	Governance					South Korea	Legislative procedure of the Infectious Disease Control and Prevention Act	N/A
Ludolph et al. (2018)	9		Communication and Public Knowledge				Hong Kong, China	533 Hong Kong residents	Quantitative Cross-sectional survey
Park et al. (2018)	7	Governance		Stigma and discrimination		Health Workers	Gyeonggi, South Korea	187 nurses	Quantitative Cross-sectional exploratory
de Zwart et al. (2009)	20		Communication and Public Knowledge				Europe (5 countries) and Asia (3 countries)	3436 respondents interviewed over the phone	Quantitative Survey
Eichelberger (2007)	3			Stigma and discrimination			New York City, NY	37 community members of New York’s Chinatown	Qualitative Participant observation
Straus et al. (2004)	29					Health Workers	Toronto, Canada	14 physicians in specialties involving SARS patients	Qualitative interviews
Des Jarlais et al. (2005)	24		Communication and Public Knowledge	Stigma and discrimination			New York City, NY	1832 New York City residents	Quantitative Phone interview
Voeton et al. (2009)	27		Communication and Public Knowledge				United Kingdom and The Netherlands	299 British and Dutch Chinese people’s knowledge of SARS compared to 804 British and Dutch non-Chinese people	Quantitative Computer assisted phone survey
Yoo et al. (2016)	10		Communication and Public Knowledge		Social Compliance		South Korea	1000 Korean adults 19 or older of nationally represented demographics	Quantitative Online survey
Oh et al. (2020)	28		Communication and Public Knowledge				South Korea	400 individuals chosen based on age, gender, and region	Quantitative Online survey
Cava et al. (2005)	25			Stigma and discrimination			Toronto, Canada	21 English speaking individuals exposed to SARS during the outbreak in Toronto	Qualitative
Qiu et al. (2018)	30			Stigma and discrimination			China	Literature review, document analysis, and 26 in-depth interviews with key stakeholders were conducted.	Qualitative case study
Siu (2008)	4			Stigma and discrimination			Hong Kong, China	200 members of a self-help group for SARS victims, 30 participated in in-depth semi-structured interviews.	Qualitative participant observation
Gautret et al. (2016)	22				Social Compliance		Middle East	Large scale surveillance study of people traveling back to the Middle East.	Quantitative Surveillance study
Affonso et al. (2003)	2					Health Workers	Toronto, Canada	Reviewed public health records, governmental and non-governmental health statements, initial epidemiological research done on SARS, and firsthand experiences of healthcare systems.	N/A
Crawford et al. (2016)	26	Governance					N/A	21 individuals who were key responders in recent epidemics participated in interviews.	Qualitative Non-systematic review
Alhomoud and Alhomoud (2017)	23				Social Compliance		Mecca, Saudi Arabia	257 pilgrims that participated in Hajj 2015 who were 18+ and spoke English or Arabic	Quantitative Cross-sectional study

## Data Availability

The data presented in this study are openly available in the electronic bibliographic databases PubMed, CINAHL, PsycINFO, and Web of Sciences.
